# Risk and Protective Factors in Schizotypy, Mistrust and Anomalous Perceptual Experience: a Network Analysis

**DOI:** 10.1192/j.eurpsy.2025.1466

**Published:** 2025-08-26

**Authors:** P. Do Lan, M. Schärer, M. Müller, V. Ajdacic-Gross, W. Rössler, B. Kleim, A. Theodoridou

**Affiliations:** 1 Psychiatry, Psychotherapy and Psychosomatics, Psychiatry University Hospital of Zurich; 2 Department Experimental Psychopathology and Psychotherapy, University of Zurich; 3 The Zurich Program for Sustainable Development of Mental Health Services (ZInEP), Psychiatry University Hospital of Zurich, Zurich, Switzerland; 4 Department of Psychiatry and Psychotherapy, Charité University Medicine, Berlin, Germany; 5 Faculty of Medicine, University of Zurich, Zurich, Switzerland

## Abstract

**Introduction:**

A resilience-based approach should be more integrated in order to get a greater understanding of the psychopathological patterns and derive prevention or intervention implications from this (Kalisch *et al. Nat Hum Behav* 2019; 1(11) 784-790). In subthreshold psychopathology, so far there is a growing body of research focusing on potential risk and protective factors while most of these studies are following an isolated focus on either of those factors. Or are using statistical methods that are not often considered the dynamic interplay of those variables (Pereira-Morales *et al.* J Ment Health 2019; 28(2) 153-160; Schäfer *et al.* Transl Psychiatry 2023; 13(1) 328). Scruitinizing the dynamic patterns enables the network approach. Mental disorders can be conceived as a complex network, involving a dynamic interplay between symptoms and protective factors (Boorsboom *et al*. Nat Rev Methods Primers 2021; 1:58*).*

**Objectives:**

This study investigates the role of risk and protective factors in relation to subthreshold psychosis like-experience symptoms (schizotypy, mistrust and anomalous perceptual experience) in a network structure.

**Methods:**

This cross-sectional analysis of the prospective longitudinal ZInEP Epidemiology Survey included n = 632 participants (general population), aged 20-41 years. Dynamic relationships between potential risk factors (child hood trauma, maladaptive coping, self-stigma, perceived stress, chronic stress), potntial protective factors (adaptive coping, self-efficacy, optimism, self-confidence, self-control, spirituality) and psychopathology (schizotypy, mistrust, anomalous perceptual experience) are investigated using network analysis at baseline.

**Results:**

- negative association of schizotypy with optimism and self-control
- negative association between mistrust and self-control
- positive association of schizotypy with perceived and chronic stress, maladaptive coping and childhood trauma
- perceived stress highly negatively assiociated with optimism and self-efficacy
- maladaptive coping as a bridge from potential protective factors to perceived and chronic stress and schizotypy

**Image 1:**

**Figure:**
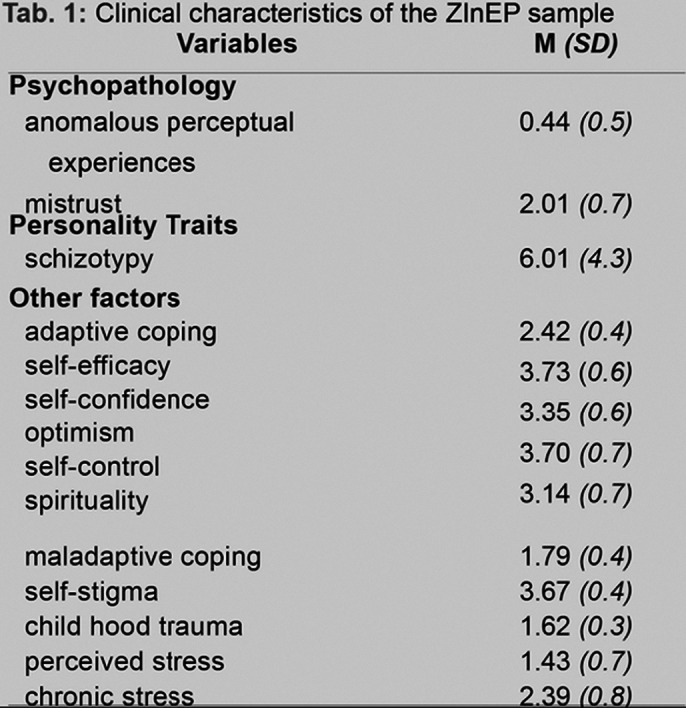

**Image 2:**

**Figure:**
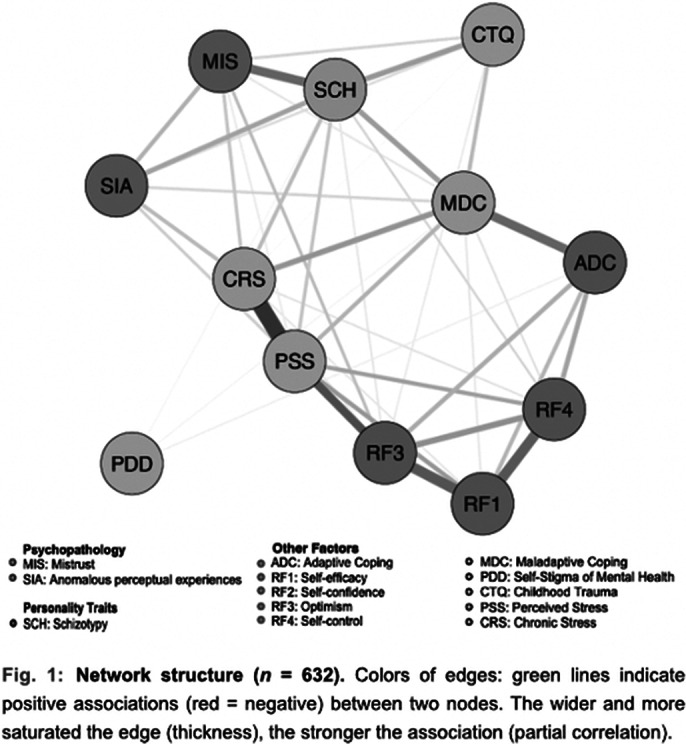

**Conclusions:**

- optimism and self-control as protective factors for schizotypy and mistrust
- perceived and chronic stress, maladaptive coping and childhood trauma as risk factors associated with all psychopathological symptoms
- protective factors might have more an indirect impact over risk factors on symptoms
- interventions for optimism and self-control might reduce stress

**Disclosure of Interest:**

None Declared

